# Genetic diversity of *Plasmodium vivax* clinical isolates from southern Pakistan using *pvcsp* and *pvmsp1* genetic markers

**DOI:** 10.1186/1475-2875-12-16

**Published:** 2013-01-11

**Authors:** Afsheen Raza, Najia K Ghanchi, Ali M Thaver, Sana Jafri, Mohammad A Beg

**Affiliations:** 1 Department of Pathology and Microbiology, Aga Khan University, Stadium Road, PO Box 3500, Karachi, 74800, Pakistan; 2 Aga Khan Medical College, Stadium Road, PO Box 3500, Karachi, 74800, Pakistan

**Keywords:** Malaria, *Plasmodium vivax*, Pakistan, Genetic diversity, Population structure, Circumsporozoite protein, Merozoite surface protein 1

## Abstract

**Background:**

*Plasmodium vivax* is the prevalent malarial species accounting for 70% of malaria burden in Pakistan; however, there is no baseline data on the circulating genotypes. Studies have shown that polymorphic loci of gene encoding antigens *pvcsp* and *pvmsp1* can be used reliably for conducting molecular epidemiological studies. Therefore, this study aimed to bridge the existing knowledge gap on population structure on *P*. *vivax* from Pakistan using these two polymorphic genes.

**Methods:**

During the period January 2008 to May 2009, a total of 250 blood samples were collected from patients tested slide positive for *P*. *vivax*, at the Aga Khan University Hospital, Karachi, or its collection units located in Baluchistan and Sindh Province. Nested PCR/RFLP was performed, using *pvcsp* and *pvmsp1* markers to detect the extent of genetic diversity in clinical isolates of *P*. *vivax* from southern Pakistan.

**Results:**

A total of 227/250 (91%) isolates were included in the analysis while the remainder were excluded due to negative PCR outcome for *P*.*vivax*. *Pvcsp* analysis showed that both VK 210 (85.5%, 194/227) and VK 247 type (14.5%, 33/227) were found to be circulating in *P*. *vivax* isolates from southern Pakistan. A total of sixteen and eighty-seven genotypes of *pvcsp* and *pvmsp*-*1* were detected respectively.

**Conclusion:**

This is the first report from southern Pakistan on characterization of *P*. *vivax* isolates confirming that extensively diverse *pvcsp* and *pvmsp1* variants are present within this region. Results from this study provide valuable data on genetic diversity of *P*. *vivax* that will be helpful for further epidemiological studies.

## Background

*Plasmodium vivax* malaria is an important public health problem causing an estimated 80–300 million clinical infections annually [1]. In Pakistan it is the common malarial species, contributing to 70% of the malaria burden [2]. Although highly prevalent, *P*. *vivax* has received scant scientific attention in Pakistan. Thus, paucity of baseline data exists on various aspects of *P*. *vivax*, such as population structure and drug resistance patterns.

Molecular epidemiological studies on genetic diversity of *P*. *vivax* have been based mainly on single copy polymorphic genes which code for parasite surface antigens such as gametocyte antigen-1 (*pvgam*-*1*), circumsporozoite protein (*csp*), merozoite surface protein-1 (*msp*-*1*) and merozoite surface protein 3 alpha (*msp 3α*) [3]. Briefly, CSP is a protein involved in binding of sporozoite to liver cells [3-6]. It comprises of central domain of tandem repeated sequences flanked by two non-repeated conserved sequences [7-10]. Two types of repeat elements, either VK210 or VK247 types are detected in clinical isolates of *P*. *vivax* and thus *csp* serves as a useful tool for genotyping [11,12]. Similarly, MSP-1 protein is responsible for red cell invasion by the parasite [13]. Structurally, it is a mosaic organization of 13 regions of inter-allele conserved and extensively polymorphic variable blocks. Three main regions of sequence divergence, at variable blocks 2, 6–8 and 10 have been revealed through studies [14]. Size polymorphisms in the respective blocks can be detected through PCR, thus allowing detection of genetically distinct *msp 1* populations within a region [15].

Molecular studies on circulating genotypes provide an insight into transmission intensity and parasite rate variation within a region. Usually, in areas of high malaria transmission, extensive genetic diversity is observed [16,17]. However, no study as yet has been reported on genetic diversity and transmission intensity of *P*. *vivax* from southern Pakistan. This study aims to provide baseline epidemiological data on circulating *pvcsp* and *pvmsp 1* allelic variants from three malaria-endemic areas of southern Pakistan, i e, Karachi, Baluchistan and Sindh.

## Methods

### Study design, settings and ethical considerations

A descriptive study for detection of *P*. *vivax* allelic variants was carried out between January 2008 and May 2009. A total of 250 patients, presenting with microscopy confirmed asexual *P*. *vivax* mono-infection at Aga Khan University Hospital, Karachi or its collection units located in Sindh and Baluchistan Provinces were enrolled in the study. The study was approved by the Ethical Review Committee of Aga Khan University Hospital, Karachi. Informed consent was obtained from enrolled patients or in the case of children, from their parents/legal guardians. Pregnant women, children under three years, and those not consenting to participate were excluded from the study.

### Blood collection and microscopy

Approximately 2 ml of intravenous blood sample in EDTA tube was collected. Initial presence of malaria parasites was established by Leishman’s staining [18] while further species identification was determined by Giemsa staining of thick and thin blood smears. Patients with microscopically confirmed *P*. *vivax* infection were included in the study. The remaining blood was stored at -80°C until DNA extraction.

### DNA extraction and amplification

DNA was extracted from 200 μl of whole blood using QiAamp DNA Mini Kit (QIAGEN, USA) according to manufacturer’s instructions. Nested PCR was performed for central repetitive domain (VK 210 or VK 247 type) of *pvcsp* and variable blocks 2, 6–8 and 10 (designated as F1, F2 and F3 respectively) of *pvmsp1* described previously [15]. Briefly, all amplification reactions were carried out in a total volume of 20 μl in the presence of 10 Mm Tris–HCl pH 8.3, 50 Mm KCL, 250 nM of each specific oligonucleotide primers,125 μM of each of the four dNTPs and 0.4 units of Taq polymerase (Invitrogen, USA). One μl of genomic and amplified DNA was used for primary and nested amplification reactions respectively. The cycling parameters were as follows: initial denaturation at 95°C for 5 min, followed by annealing at defined temperatures for each primer pair for 2 min, extension at 72°C for 2 min, denaturation at 94°C for 1 min and final annealing step followed by extension for 5 min [15]. Analysis of PCR products was done by gel electrophoresis. Briefly, amplified product was loaded on 2% agarose gels alongside 100 bp molecular weight marker, separated in TBE buffer by electrophoresis, stained with ethidium bromide and visualized under UV transilluminator (Gel Doc, Bio-Rad, Hercules, USA).

### PCR/RFLP

PCR/ RFLP was performed for *pvcsp* and *pvmsp 1* F2 fragment as described previously [15]. Briefly, amplified products were digested separately with restriction enzymes, *Alu I* and *Bst NI* for *pvcsp* and *Alu1* and *Mnl1* for *pvmsp1* F2 in a total volume of 20 μl for 3 hrs according to the supplier’s specifications (New England Biolabs Inc., UK) The digested product was analysed on 1.5% agarose gel following ethidium bromide staining.

### Identification of recurrent infections using *pvmsp1* marker

For identification of recrudescence, relapse/new infections, *pvmsp1* amplified products from recurrence samples was run side by side on agarose gel alongside 100 bp molecular weight marker. Band sizes were determined using Quantity one software (Biorad, Hercules, USA). Recrudescence, relapse/ new infections were identified on the basis of differences/similarities in genotypes [19-21].

### Statistical analysis and allele detection

Data was entered in Microsoft Excel and exported to SPSS 19.0 for analysis. Arithmetical means and medians were calculated, where applicable, for all continuous baseline demographic variables. *Pvcsp* repeat types were detected on the basis of digestion with the respective restriction enzymes [15]. For determination of size polymorphisms, Quantity One software (Biorad, Hercules, USA) was used. Further analysis of number of genotypes and size polymorphisms in *pvmsp1* F1, F2, F3 and *pvcsp* was done by grouping the electrophoresis band sizes into “bins” differing by 20 bp. The median genotype of each fragment was identified. The absolute size of the identified median band +/− 10 bp formed the initial bin. Thereafter, each 20 bp interval below and above the median band were defined as representing a distinct genotype [22,23].

## Results

### Baseline demographic data

A total of 250 patients with microscopy-confirmed *P*. *vivax* mono-infection were enrolled from Karachi, Baluchistan and rural areas of Sindh Province. Out of these, 227 (91%) were included in the analysis while the remaining 23 (9.2%) were excluded due to negative PCR outcome for *P*. *vivax*. Baseline demographics of patients included in the analysis are presented in Table 1.

**Table 1:** Baseline characteristics of enrolled patients

	**All (n** **=** **227)**	**Karachi (n** **=** **183)**	**Baluchistan (n** **=** **23)**	**Sindh (n** **=** **21)**
**Age**				
5-15 years	43	30	3	10
> 15 years	184	153	20	11
**Sex**				
Male	167	131	20	16
Female	60	52	3	5

### Distribution of *pvcsp* repeats types

PCR RFLP analysis of *pvcsp* genetic marker revealed that out of 227 isolates, 194 (85.5%) and 33 (14.5%) harboured parasite populations carrying VK 210 and VK 247 repeat types respectively while mixed genetic infection (both VK210 and VK 247) was observed in four (1.8%) of the isolates. Base pair ranges of VK210 were 620-800bp and 680-820bp for VK247 type. A total of 16 genotypes (9 for VK210 and 7 for VK 247) were detected on the basis of size variation in the repeat types.

**Figure 1:**
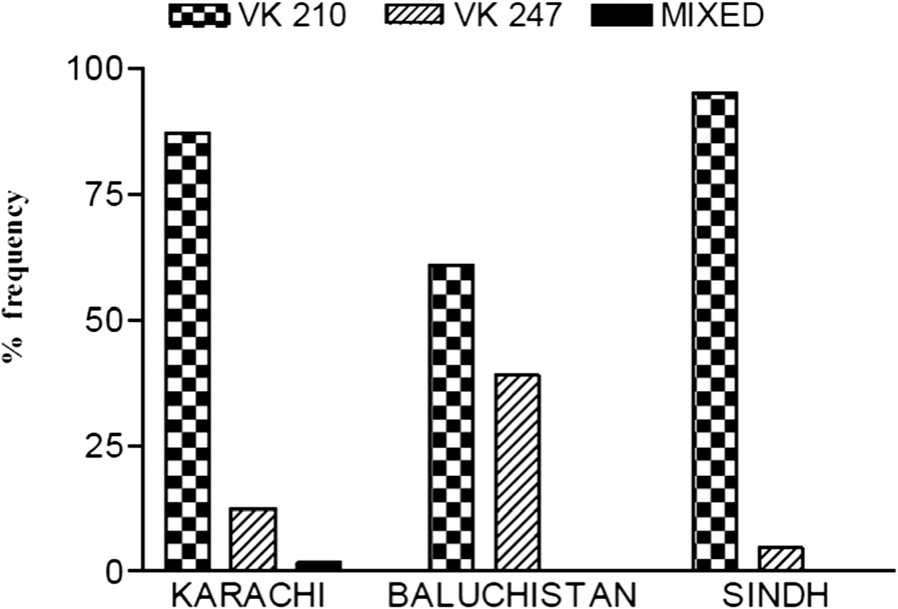
**Distribution of *pvcsp *types in Karachi**, **Baluchistan and Sindh province.**

VK 210 type was found to be predominant in Karachi (87.4%, 160/183), Baluchistan (60.8%, 14/23) and Sindh (95.2%, 20/21) while the percent positivity of VK 247 type was found to be 12.5% in Karachi, 39% in Baluchistan and 4.8% in Sindh Furthermore, mixed genetic infection was observed only in Karachi (2.2%)(Figure 1).

### *Pvmsp 1* allelic diversity

Length polymorphism was assessed within three main regions of sequence divergence (F1, F2 and F3) in *pvmsp 1* in all three study areas. Thirty-six genotypes were observed in Karachi, 28 in Baluchistan and 23 in Sindh. Thus, a total of 87 distinct genotypes were observed in southern Pakistan. The geographical distribution of F1, F2 and F3 variants with base pair ranges is given in Table 2. Mixed genotype infections were observed frequently in F1, F2 and F3 fragments in Karachi, F2 and F3 fragments in Baluchistan and only in F3 fragment in Sindh (Table 2). The mean multiplicity of infection was calculated to be 1.1 for Karachi and 1.2 for Baluchistan and Sindh.

**Table 2:** **Geographical distribution of *pvmsp 1 *genotypes with base pair ranges**

	**Base pair ranges**	**No. of genotypes**	**No. of mixed variants**
**Karachi**			
F1	320-550	10	6
F2	900-1270	16	4
F3	200-400	10	11
**Baluchistan**			
F1	330-550	8	0
F2	960-1270	14	1
F3	200-320	6	3
**Rural Sindh**			
F1	340-460	5	0
F2	930-1190	11	0
F3	200-350	7	5
**Total no. of genotypes**		87	

F1 = Fragment 1 representing variable block 2, F2 = Fragment 2 represents variable block 6–8, F3 = Fragment 3 representing variable block 10.

For *pvmsp1* F2 fragment, a total of 88 isolates (Karachi = 64, Baluchistan = 12, Sindh = 12) were analysed by RFLP for further distinction of allelic variants. In Karachi, two Mnl 1 and four Alu 1 patterns, in Baluchistan one *Mnl 1* and three *Alu 1* patterns while in Sindh, one *Mnl 1* and one *Alu 1* pattern were observed. Therefore, combining data from both size polymorphism and RFLP, a total of 28 distinct F2 variants could be differentiated in Karachi, Baluchistan and Sindh respectively.

### Identification of recurrent infections

A total of four cases, all from Karachi, were identified as recrudescence or relapse/new infections using *pvmsp1* marker. One case, re-admitted after 1month, revealed the same genotype as that identified earlier while the remaining three cases, re-infected after 6–8 months showed different genotype than the earlier samples and was thus regarded as relapse/new infection.

## Discussion

This is the first study on genetic diversity of *P*. *vivax* clinical isolates from southern Pakistan and the results reveal that extensively diverse *P*. *vivax* populations are present in this region. By using *pvcsp* genetic marker, both VK210 and VK 247 types were found in parasite population from southern Pakistan with VK210 being the predominant type. However, the percent positivity of VK 247 type (14.5%) is close to the findings from Herat, Afghanistan (10.4%) [24] and south-eastern areas of Iran (17.5%) [25] but different from that reported from Northern Belt (FATA region) of Pakistan (2.7%) [26]. With respect to study areas, a similar pattern was observed with VK 247 type percent positivity being high in Baluchistan (39%) and Karachi (12.5%) but not in Sindh Province (4.8%). This sharp difference in the frequency of VK 247 type between southern and northern regions of Pakistan and between the respective study areas may be attributed to a number of factors such as co distribution of various vector species and subsequently increased susceptibility of these species to infection by VK 247 type, sampling biases, migration rates, host immune pressure and regional temporal fluctuations [25,27]. It is possible that these factors, either alone or in conjunction with each other may be allowing gradual natural selection of VK 247 type, particularly in Baluchistan and Karachi.

A total of 16 allelic variants of *pvcsp* types were observed on the basis of size polymorphisms which is consistent with 15-21varients types observed in previous studies [5,28]. However, other studies from Thailand and India reported ten and three variants respectively [15,27]. Studies suggest that specific variant type infecting an individual may influence induced immune responses [29-31]. Therefore, detection of diverse VK 210 and VK 247 types in this study may serve as a baseline for conducting further studies to understand the role of these variants in host response.

With *pvmsp1*, a total of 87 distinct genotypes could be distinguished through size polymorphisms in southern Pakistan (Table 2). With respect to F1, F2 and F3 fragments, 23, 41 and 23 allelic variants were detected in the three study areas respectively, indicating that extensive polymorphism has accumulated in *msp*-*1* gene, particularly in F2 fragment. This result is inconsistent with that reported from Thailand and India [15,27], in which the F2 fragment was found to be poorly polymorphic, with two allelic variants detected through size polymorphism and further distinction of F2 variants required RFLP analysis. The results of this investigation revealed that the F2 fragment in *P*. *vivax* isolates from Pakistan, is extensively polymorphic and can be genotyped easily using PCR only with no further allelic distinction through RFLP. However, further studies on F2 fragment from different areas of Pakistan and with larger sample size, is required to corroborate these results. Moreover, mixed genotype infections were observed frequently in all the three *msp*-*1* fragments in Karachi, F2 and F3 fragments in Baluchistan and only in F3 fragment in Sindh (Table 2). The F3 mixed variants were observed predominantly in all three study areas indicating the predisposition of this block to mixed infections. Such high rate of mixed infections and prevalence of genetically diverse isolates signifies that *pvmsp*-*1* gene is under selective pressure for its survival and transmission in Pakistan. This finding is important with respect to transmission intensity and surveillance of *P*. *vivax* in southern Pakistan.

The ability to detect genetically diverse variants in all the three fragments of *pvmsp1* signifies that this marker can serve useful in identification of individual’s infections. In *P*. *falciparum*, recrudescence is identified when the genotype pattern for the two paired samples of a study participant is the same or any bands are shared between the two samples, while a new infection is indicated when the patterns are completely different [32]. Studies suggest that similar strategy can be used for identification of relapses/recrudescence in *P*. *vivax*. However, distinction between relapse and recrudescence is difficult due to the high variability in the genotypes of parasite during relapse/recurrence/new infections [19-21]. In this study, three relapses and one recrudescence case has been identified on the basis of differences/similarities in the *pvmsp*-*1* genetic marker. However, extensive polymorphism in *P*.* vivax* and small number of relapse samples collected during this study suggests interpretation of recurrent infection with caution.

Molecular characterization on population structure of *P*. *vivax* isolates from southern Pakistan has been explored for the first time in this study. The extensive polymorphism and diversity observed in *pvcsp* and *pvmsp1* genetic markers is indicative of natural selective pressure on the parasite for its survival and transmission in the region. However, further studies from different areas of Pakistan are needed to determine the prevalent genotypes and the transmission intensity of *P*. *vivax*. Results from this study can serve as a baseline for conducting studies for effective malaria control.

## Conclusion

Increased sequence variation is observed in *P*. *vivax*, signifying the high transmission rates and consequently the difficulties associated with malaria control in Pakistan.

## Abbreviations

F1: Fragment1; F2: Fragment2; F3: Fragment3; PCR: Polymerase chain reaction; pvcsp: *Plasmodium vivax* circumsporozoite protein gene; pvgam 1: *Plasmodium vivax* gametocyte gene; pvmsp 1: *Plasmodium vivax* merozoite surface protein 1 gene; pvmsp 3 α: *Plasmodium vivax* merozoite surface protein 3 *α* gene; RFLP: Restriction fragment length polymorphism.

## Competing interests

The authors declare that they have no competing interests.

## Authors’ contributions

AR performed PCR genotyping, data entry, statistical analysis and interpretation as well composed the manuscript. NKG designed, planned the study and reviewed the final draft. SJ performed data entry, DNA extractions, PCR genotyping and statistical analysis. AT participated in DNA extractions. MAB designed and planned the study, performed data analysis and interpretation and wrote the report. All authors read and approved the final manuscript.
